# Nomogram models for predicting outcomes in thyroid cancer patients with distant metastasis receiving ^131^iodine therapy

**DOI:** 10.1038/s41598-025-86169-7

**Published:** 2025-01-20

**Authors:** Shui Jin, Xuemei Ye, Ting Ye, Xinyu Chen, Jianfeng Ji, Jinyu Wang, Xin Zhu, Xiaochun Mao, Takahiro Higuchi, Heqing Yi

**Affiliations:** 1 Department of Nuclear Medicine, Zhejiang Cancer Hospital, Hangzhou, 310022 Zhejiang China; 2 Key Laboratory of Head and Neck Cancer Translational Research of Zhejiang Province, Zhejiang Cancer Hospital, Hangzhou, 310022 Zhejiang China; 3 Nuclear Medicine, Faculty of Medicine, University of Augsburg, Augsburg, Germany; 4 Medical records and statistics office, Zhejiang Cancer Hospital, Hangzhou, 310022 Zhejiang China; 5 Department of Thyroid Surgery, Zhejiang Cancer Hospital, Hangzhou, 310022 Zhejiang China; 6 Department of Nuclear Medicine and Comprehensive Heart Failure Center, University Hospital Würzburg, ZIM House A4, Oberdürrbacher Str., 697080 Würzburg, Germany; 7 Faculty of Medicine, Dentistry and Pharmaceutical Sciences, Okayama University, Okayama, Japan

**Keywords:** ^131^iodine, Activity, Distant metastasis, Iodine radioisotopes, Thyroid cancer, Thyroid cancer, Radiotherapy

## Abstract

This study aimed to establish and validate prognostic nomogram models for patients who underwent ^131^I therapy for thyroid cancer with distant metastases. The cohort was divided into training (70%) and validation (30%) sets for nomogram development. Univariate and multivariate Cox regression analyses were used to identify independent predictors for overall survival (OS) and progression-free survival (PFS). Nomograms were developed based on these predictors, and Kaplan-Meier curves were constructed for validation. Among 451 patients who were screened, 412 met the inclusion criteria and were followed-up for a median duration of 65.2 months. The training and validation sets included 288 and 124 patients, respectively. Pathological type, first ^131^I administrated activity, and lesion ^131^I uptake in lesions were independent predictors for PFS. For OS, predictors included gender, age, metastasis site, first ^131^I administrated activity, ^131^I uptake, pulmonary lesion size, and stimulated thyroglobulin levels. These predictors were used to construct nomograms for predicting PFS and OS. Low-risk patients had significantly longer PFS and OS compared to high-risk patients, with 10-year PFS rates of 81.1% vs. 51.9% and 10-year OS rates of 86.2% vs. 37.4%. These may aid individualized prognostic assessment and clinical decision-making, especially in determining the prescribed activity for the first ^131^I treatment.

**Supplementary Information:**

The online version contains supplementary material available at 10.1038/s41598-025-86169-7.

## Introduction

The management of differentiated thyroid cancer (DTC) remains a clinical challenge, especially in cases with metastases to the lung and other organs. Administration of iodine-131 (^131^I) remains the key therapeutic approach in these cases, and offers potential for disease control and improved survival^1–3^. However, the response to ^131^I therapy varies considerably among individuals owing to differences in tumor biology and patient and clinical characteristics^4–8^.

In this context, few studies have evaluated the prognostic impact of first dose activity on metastases^9–11^. The appropriate activity for treatment is currently determined based on three reference levels: (1) dose absorbed by the lesion (i.e., an absorbed dose of > 80 Gy for achieving effective treatment), (2) empirical fixed activity of administered ^131^I, and (3) maximum-tolerated activity^10–12^. Over the years, clinical guidelines have recommended activities in the range of 3.70–7.40 GBq (100–200 mCi) for the treatment of lung and other distant metastases. However, this clinically applicable range is considered to be overly broad^10,13,14^. This has necessitated the development of predictive tools (based on multiple prognostic factors) which may accurately estimate individual treatment outcomes and guide clinical decision-making. Although nomograms visually represent the relationship between relevant factors and patient prognosis in an intuitive manner, few are available for predicting prognosis after treatment with ^131^I.

To address this gap, our study aimed to develop and validate a comprehensive nomogram model. This tool, which incorporated relevant clinical and treatment-related parameters, was used to predict survival outcomes among patients with thyroid cancer who underwent ^131^I therapy for distant metastases. Detailed patient data were obtained from a single center and rigorous statistical methods were used to obtain a robust model, which could enhance prognostication and aid personalized management of these patients.

## Results

### Clinical characteristics

Among 9,014 patients who received ^131^I treatment for thyroid cancer between 2007 and 2020, 451 had pulmonary metastases; 6 died from non-thyroidal cancers, 20 developed pulmonary metastases more than 6 months after ^131^I treatment, and 13 were aged under 18 years. After excluding these patients, 412 were eligible for evaluation. Among them, 281 (68.2%) and 131 (31.8%) were female and male, respectively (female-to-male ratio of 2.15:1). The median age of the cohort was 49.0 (range: 18 to 79) years, and the duration of follow-up was 65.2 (interquartile range: 42.8-108.2) months.

Among the participants, 335 (81.3%) had isolated lung metastases and 77 (18.7%) presented with metastases to the lung and other organs (such as the bone, liver, and/or brain). The pathological types included papillary thyroid cancer (356 [86.4%]), follicular thyroid cancer (49 [11.9%]), and poorly differentiated thyroid carcinoma (7[1.7%]). The mean of first ^131^I administrated activity was 6.05 ± 1.35 (range: 1.11–9.25) GBq. On an average, each patient received 3.1 (range: 2–11) ^131^I treatments; this resulted in a mean cumulative activity of 20.99 ± 10.40 (range 9.25-80) GBq.

**Fig. 1:**
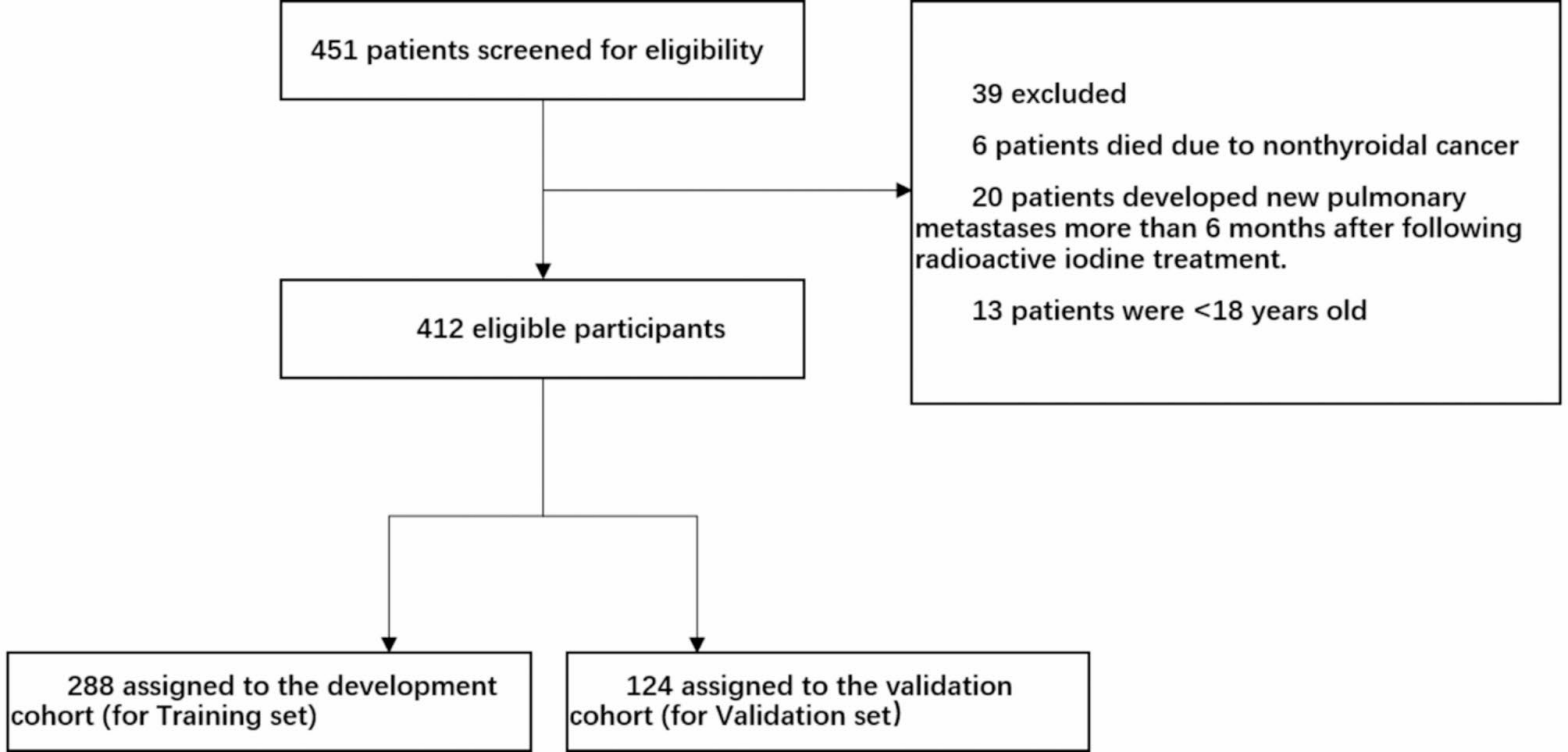
Flowchart depicting patient selection.

Among the 412 patients, 288 (69.9%) showed uptake of ^131^I by distant metastases during the first treatment. However, 124 (30.1%) showed no uptake during the first and subsequent treatment (primary non-uptake) and 112 (27.2%) demonstrated no uptake during the second or subsequent treatments (secondary non-uptake). Efficacy evaluation during subsequent treatments and follow-up showed CR, PR, SD, and PD in 27 (6.6%), 194 (47.1%), 91 (22.1%), and 100 (24.3%) patients, respectively. A total of 52 (12.6%) patients had died by the last follow-up date, and the 5- and 10-year survival rates were notably high at 92.5% and 80.0%, respectively. The study population was divided into training and validation sets having 288 (70%) and 124 (30%) patients, respectively (Fig. 1). Comparison between the training and validation sets showed no significant differences in terms of median OS and PFS (Table 1).

**Table 1:** Clinical characteristics of patients with DTC treated with ^131^I.

	ALL	Training	Validation
	*N* = 412	*N* = 288	*N* = 124
Gender			
Female	281 (68.20%)	195 (67.71%)	86 (69.35%)
Male	131 (31.80%)	93 (32.29%)	38 (30.65%)
Age			
<55.00	257 (62.38%)	177 (61.46%)	80 (64.52%)
≥55.00	155 (37.62%)	111 (38.54%)	44 (35.48%)
Pathological			
PTC^a^	356 (86.41%)	245 (85.07%)	111 (89.52%)
FTC^b^	49 (11.89%)	37 (12.85%)	12 (9.68%)
pDTC^c^	7 (1.70%)	6 (2.08%)	1 (0.81%)
T stage			
1 + 2	152 (36.89%)	115 (39.93%)	37 (29.84%)
3 + 4	198 (48.06%)	134 (46.53%)	64 (51.61%)
X	62 (15.05%)	39 (13.54%)	23 (18.55%)
N stage			
0	62 (15.05%)	48 (16.67%)	14 (11.29%)
1	326 (79.13%)	223 (77.43%)	103 (83.06%)
x	24 (5.83%)	17 (5.90%)	7 (5.65%)
Site of metastases			
Lung	335 (81.31%)	227 (78.82%)	108 (87.10%)
Lung + other	77 (18.69%)	61 (21.18%)	16 (12.90%)
First ^131^I^d^ activity (mCi)	180.00 [150.00;200.00]	180.00 [150.00;200.00]	160.00 [150.00;200.00]
^131^I uptake			
Primary iodine nonuptake	124 (30.10%)	84 (29.17%)	40 (32.26%)
Secondary iodine nonuptake	112 (27.18%)	70 (24.31%)	42 (33.87%)
Uptake	176 (42.72%)	134 (46.53%)	42 (33.87%)
Pulmonary nodule size (cm)	0.80 [0.50;1.20]	0.80 [0.50;1.20]	0.75 [0.50;1.12]
sTg^e^ (ng/mL)			
<500.00	228 (55.34%)	156 (54.17%)	72 (58.06%)
≥500.00	184 (44.66%)	132 (45.83%)	52 (41.94%)
Death			
NO	360 (87.38%)	256 (88.89%)	104 (83.87%)
YES	52 (12.62%)	32 (11.11%)	20 (16.13%)
Progression			
NO	293 (71.12%)	208 (72.22%)	85 (68.55%)
YES	119 (28.88%)	80 (27.78%)	39 (31.45%)

^a^Papillary thyroid cancer.

^b^Follicular thyroid cancer

^c^Poorly differentiated cancer cells.

^d^^131^iodine.

^e^Stimulated thyroglobulin.

## Nomogram model for OS

**Fig. 2:**
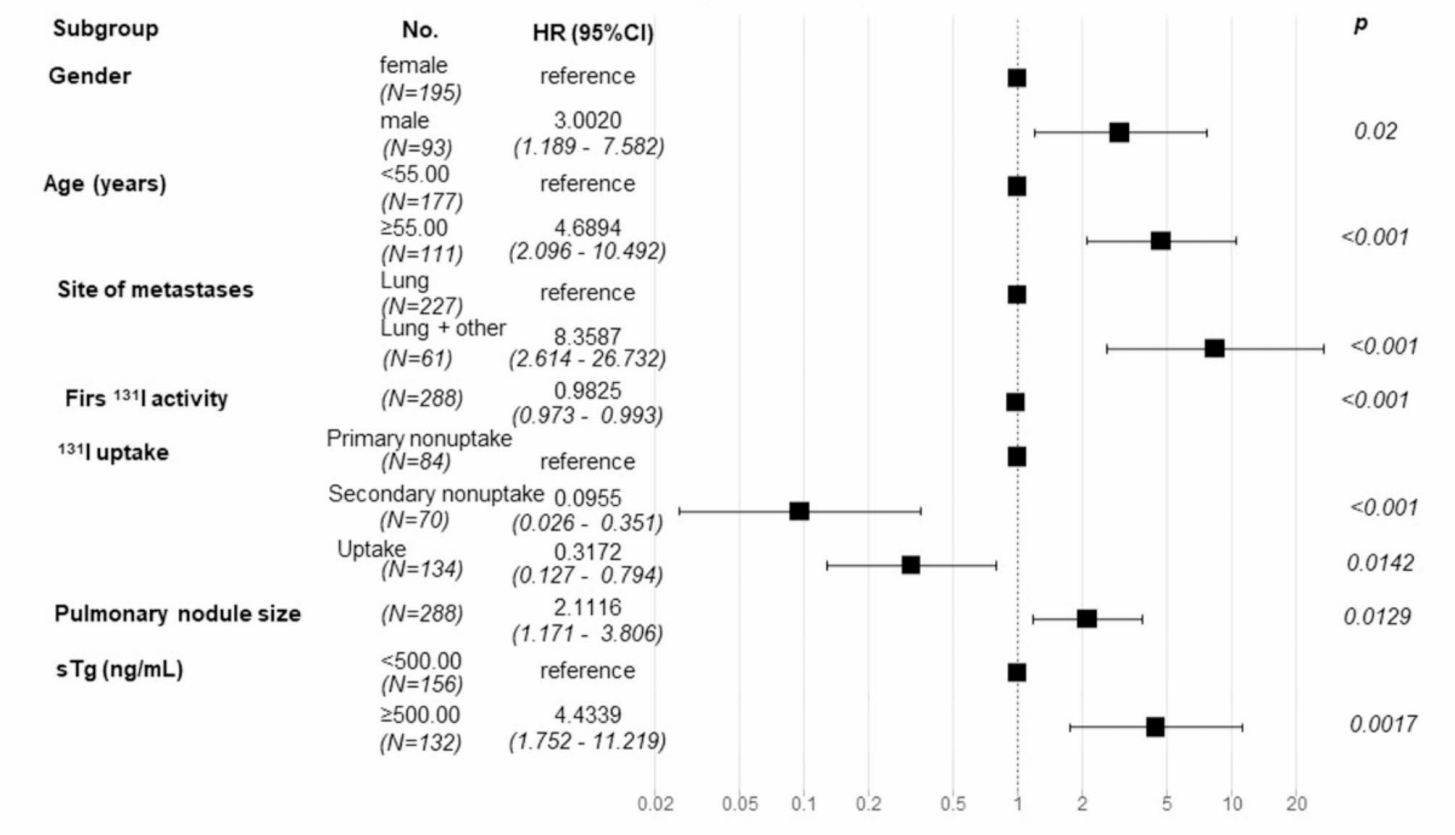
Forest plot of multivariate Cox regression analysis for OS in the training set. The predictive factors included in the multivariate analysis were selected based on the results of the univariate analysis. A Forest plot illustrating several covariates shows the survival hazard ratio for each (presented as point estimates with 95% confidence intervals [CIs] in brackets). Hazard ratios greater than 1 (positioned to the right of the reference line for each covariate) indicate worse outcomes. p-values are provided for all covariates included in the model.

Univariate analysis was first performed using OS as the outcome variable. The predictive factors selected for inclusion during multivariate analysis included gender, age, site of metastases, first ^131^I administrated activity, ^131^I uptake, pulmonary nodule size, and sTg levels (*p* < 0.05) (Table 2). Based on the results of univariate analysis, multivariate Cox regression analysis was performed using OS as the outcome variable (Fig. 2). Male gender (HR [hazard ratio] = 3.0, *p* = 0.02), age ≥ 55 years (HR = 4.7, *p* < 0.001), concurrent metastasis to the lungs and other organs (HR = 8.4, *p* < 0.001), and initial sTg levels of ≥ 500 ng/mL (HR = 4.4, *p* = 0.0017) were found to be associated with a relatively higher risk of death. The size of the largest metastatic lung nodule was directly proportional to the risk of death (HR = 2.1, *p* = 0.0129). Conversely, the first ^131^I administrated activity was inversely associated with the risk of death (HR = 0.9825, *p* < 0.0001). In terms of ^131^I uptake by the metastatic lesions, the patients with secondary non-uptake (HR = 0.0955, *p* < 0.0017) or continued uptake after multiple treatments (HR = 0.3172, *p* = 0.0142) offered significantly better survival than patients with primary non-uptake.

**Table 2:** Univariate Cox proportional hazard regression analysis for OS and PFS of distance metastatic thyroid cancer patients in the training set.

variable	OS		PFS	
	HR(95%CI)	*p*	HR(95%CI)	*p*
Gender				
Female				
Male	2.106 (1.051, 4.221)	0.036	1.324 (0.842, 2.083)	0.224
Age				
< 55.00				
≥ 55.00	5.602 (2.571, 12.207)	< 0.001	1.746 (1.126, 2.708)	0.013
Pathological				
PTC^a^				
FTC^b^	1.556 (0.595, 4.066)	0.368	0.663 (0.305, 1.443)	0.300
pDTC^c^	2.370 (0.318, 17.637)	0.399	4.125 (1.501, 11.335)	0.006
T stage				
1 + 2				
3 + 4	1.603 (0.729, 3.527)	0.241	0.814 (0.513, 1.292)	0.382
X	0.772 (0.209, 2.853)	0.698	0.584 (0.271, 1.257)	0.169
N stage				
0				
1	0.661 (0.267, 1.632)	0.369	1.130 (0.608, 2.101)	0.699
x	1.173 (0.292, 4.709)	0.822	2.121 (0.866, 5.191)	0.100
Site of metastases				
Lung				
Lung + other	3.329 (1.635, 6.778)	0.001	1.341 (0.808, 2.224)	0.257
First ^131^I^d^ activity (mCi)	0.990 (0.981, 1.000)	0.043	0.991 (0.986, 0.996)	0.001
^131^I uptake				
Primary iodine nonuptake				
Secondary iodine nonuptake	0.461 (0.178, 1.191)	0.110	0.487 (0.271, 0.875)	0.016
Uptake	0.406 (0.185, 0.889)	0.024	0.417 (0.254, 0.686)	0.001
Pulmonary nodule size (cm)	1.939 (1.196, 3.145)	0.007	1.235 (0.882, 1.728)	0.219
sTg^e^(ng/mL)				
< 500.00				
≥ 500.00	3.343 (1.544, 7.238)	0.002	0.804 (0.514, 1.258)	0.339

^a^Papillary thyroid cancer.

^b^Follicular thyroid cancer.

^c^Poorly differentiated cancer cells.

^d^^131^iodine.

^e^Stimulated thyroglobulin.

**Fig. 3:**
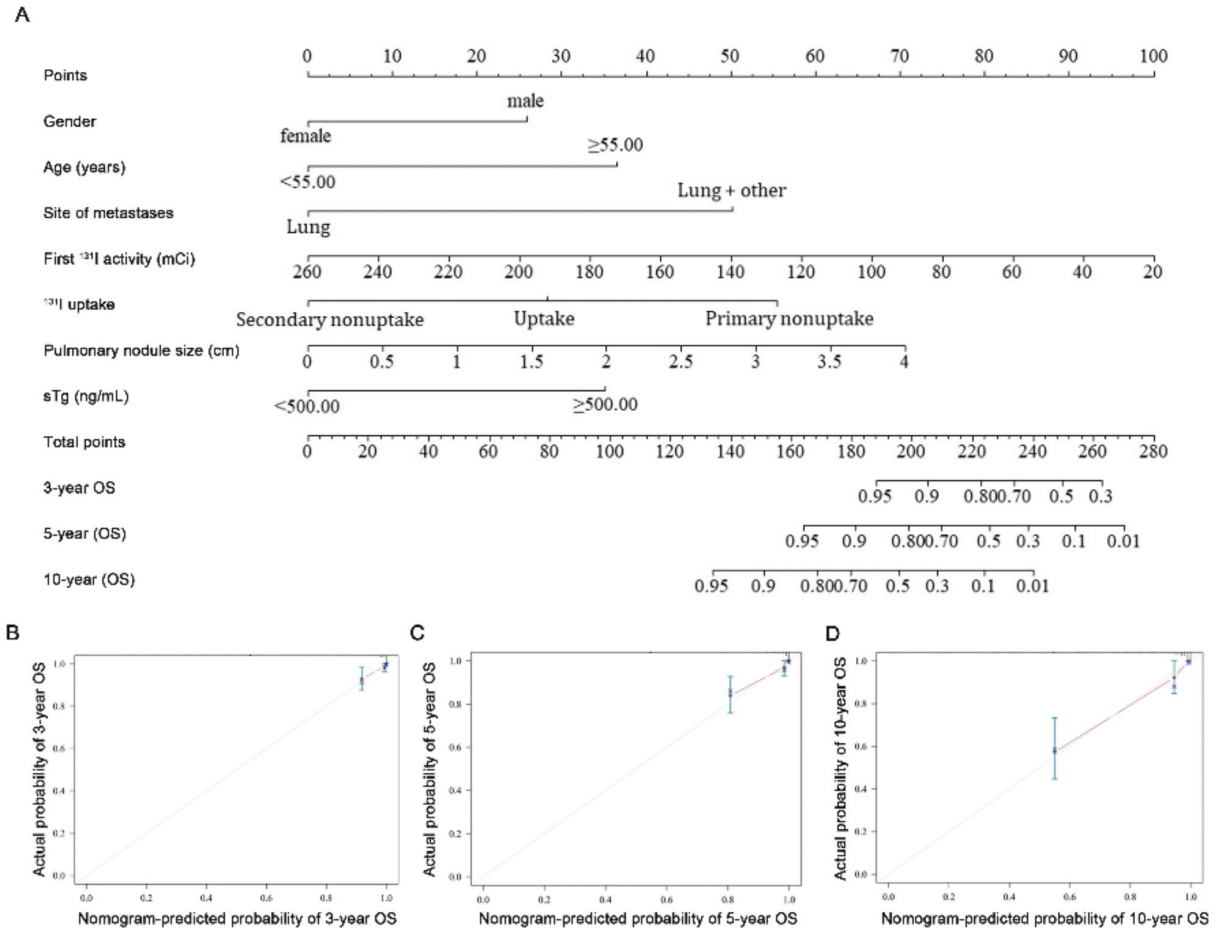
Calibration curves and nomogram for OS. Nomogram for predicting the probability of OS at 3, 5, and 10 years (**A**). Each clinical characteristic is assigned a specific point value, indicated in the top row of the nomogram. A value of 0 points is assigned if the characteristic is absent, while its presence yields a certain number of points determined by the nomogram function. These points are then summed to produce a total score. The total score is used to estimate the corresponding 3-, 5-, and 10-year OS probabilities. Calibration plots for 3 years (**B**), 5 years (**C**), and 10 years (**D**) demonstrate the concordance between predicted and observed OS probabilities. The x-axis represents nomogram-predicted OS probabilities, while the y-axis reflects observed OS probabilities. The diagonal dashed line indicates ideal calibration, where predicted outcomes closely align with observed results.

A prediction nomogram model was constructed based on the independent predictors of OS (Fig. 3A). The calibration plot demonstrated consistency between the predicted and the observed values for 3-, 5-, and 10-year OS (Fig. 3B–D). The C-index value for the model was 0.877 (95% confidence interval: 0.821, 0.933); the validation model demonstrated a similar value of 0.818 (95% confidence interval: 0.742, 0.893).

## Nomogram model for PFS

**Fig. 4:**
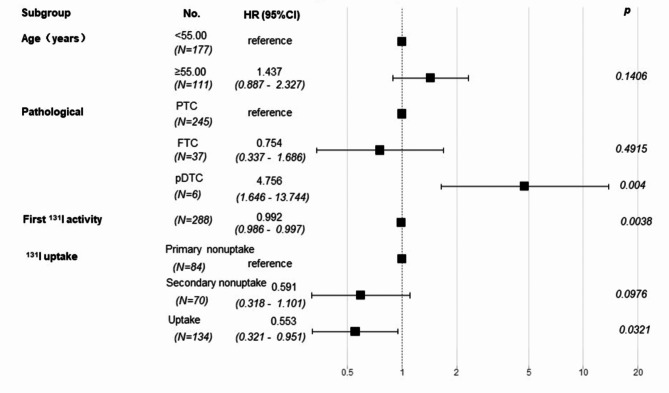
Forest plot of multivariate Cox regression analysis for PFS in the training set. The predictive factors included in the multivariate analysis were selected based on the results of the univariate analysis. A Forest plot illustrating several covariates shows the PFS hazard ratio for each (presented as point estimates with 95% CIs in brackets). Hazard ratios greater than 1 (positioned to the right of the reference line for each covariate) indicate worse outcomes. p-values are provided for all covariates included in the model.

The predictors in the model for PFS were selected using univariate analysis; these included age, pathological type, first ^131^I administrated activity, and ^131^I uptake. Multivariate Cox regression analysis (*p* < 0.05) was performed using PFS as the outcome variable (Fig. 4). An increase in the activity of the first ^131^I administrated activity was found to confer a protective effect on PFS (HR = 0.992, *p* = 0.0038). Patients with poorly differentiated cancers showed a relatively higher risk of disease progression (HR = 4.8, *p* = 0.004). Retention of ^131^I uptake by metastatic lesions after multiple treatments conferred significant PFS advantage compared to primary non-uptake (HR = 0.553, *p* = 0.0321).

**Fig. 5:**
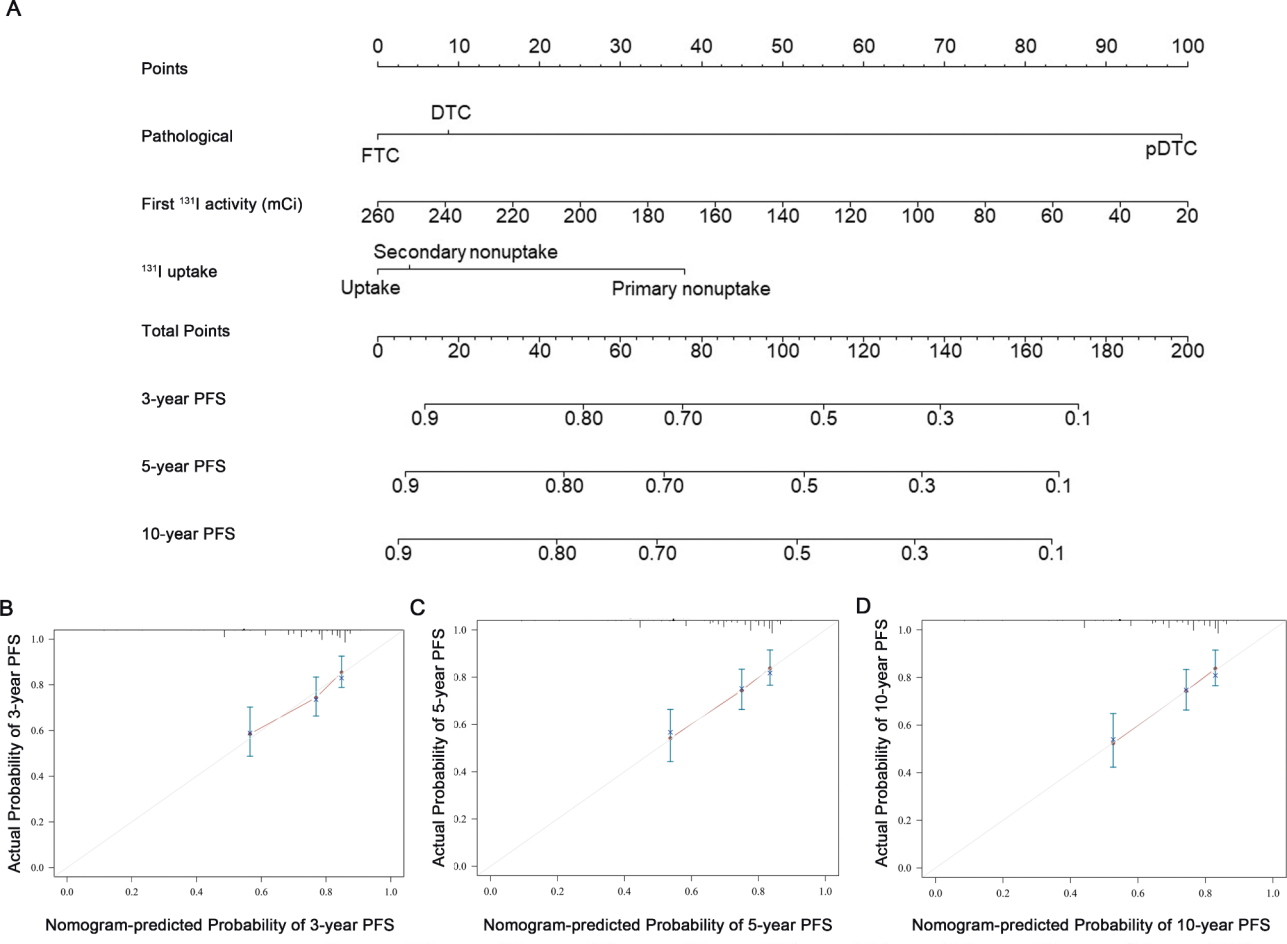
Calibration curves and nomogram for PFS. Nomogram for predicting the probability of PFS at 3, 5, and 10 years (**A**). Each clinical characteristic is assigned a specific point value, indicated in the top row of the nomogram. A value of 0 points is assigned if the characteristic is absent, while its presence yields a certain number of points determined by the nomogram function. These points are then summed to produce a total score. The total score is used to estimate the corresponding 3-, 5-, and 10-year PFS probabilities. Calibration plots for 3 years (**B**), 5 years (**C**), and 10 years (**D**) demonstrate the concordance between predicted and observed PFS probabilities. The x-axis represents nomogram-predicted PFS probabilities, while the y-axis reflects observed PFS probabilities. The diagonal dashed line indicates ideal calibration, where predicted outcomes closely align with observed results.

A nomogram prediction model was subsequently constructed based on the independent predictors (Fig. 5A). The calibration curve demonstrated strong concordance between the predicted and observed values for 3-, 5- and 10-year PFS (Fig. 5B–D). The C-index value for the model was 0.646 (0.584, 0.709); the validation model demonstrated a similar value of 0.692 (0.613, 0.770).

## Survival curves

**Fig. 6:**
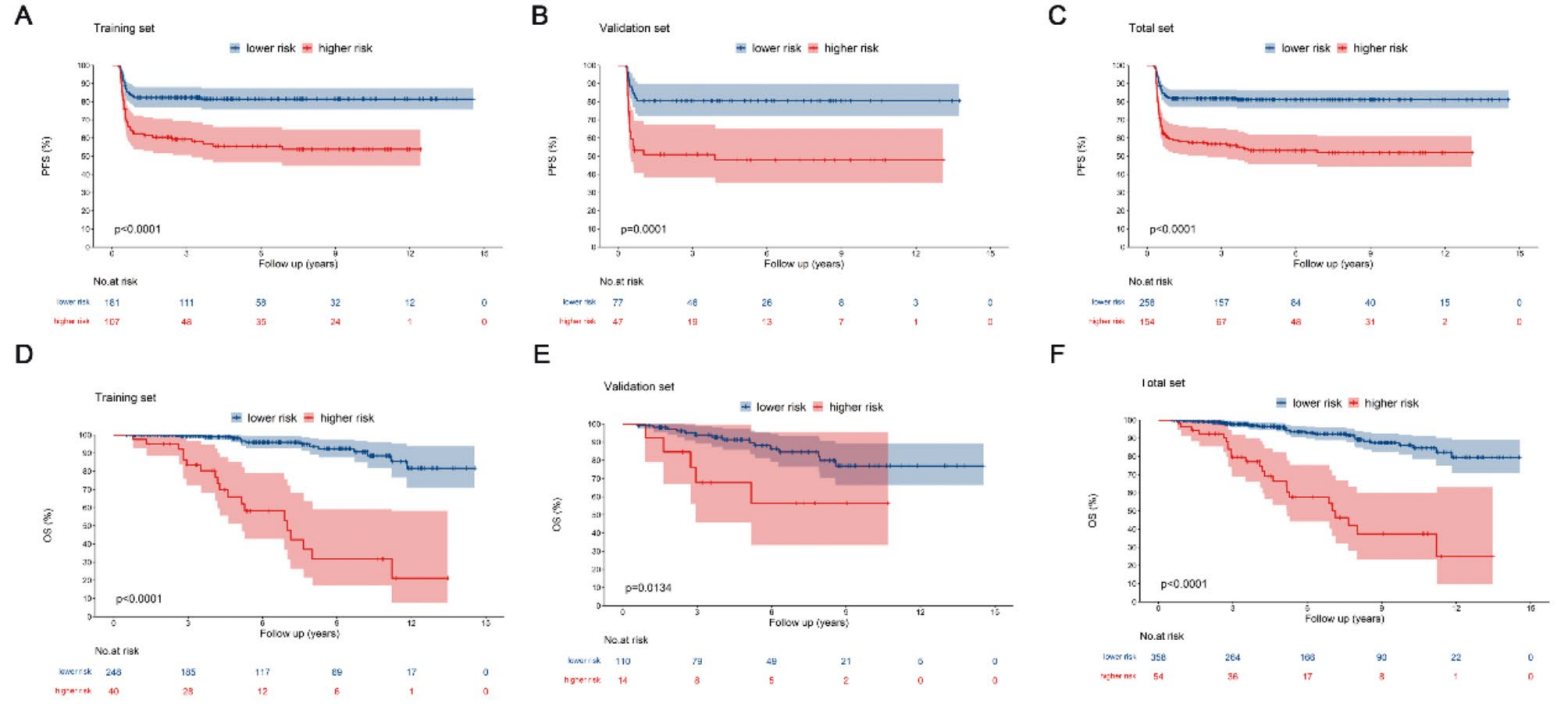
Kaplan-Meier curves of high- and low-risk patient groups, stratified based on cutoff values determined by the nomogram scores. PFS curves for the training set (**A**), validation set (**B**), and complete set (**C**), stratified into low- and high-risk groups based on optimal cutoff points. OS survival curves for the training set (**D**), validation set (**E**), and complete set (**F**), stratified into low- and high-risk groups based on optimal cutoff points.

The cutoff values for OS and PFS, as derived from calculations using the training set, were found to be 191 and 67, respectively. The subjects were grouped into high- and low-risk groups based on these values; the Kaplan-Meier curves for the groups are shown in Fig. 6. Significant differences were observed between the high- and low-risk groups across the training, validation, and complete sets in terms of 3-, 5-, and 10-year OS and PFS. The low-risk group exhibited significantly prolonged OS and PFS (Fig. 6, Table S1, *p* < 0.05).

## Discussion

Our retrospective single-center study evaluated prognostic factors in patients undergoing ^131^I therapy for thyroid cancer with distant metastases and developed a nomogram for visualizing these findings. As a first-line treatment modality following total thyroidectomy, ^131^I has demonstrated effective disease control and survival benefits^1–3,15^. However, the literature on prognostic factors and outcomes remains inconsistent, and the role of ^131^I therapy continues to be debated even after nearly 80 years of clinical use^7,8^. Variability in prognostic factors across studies may be due to differences in patient populations, analyzed factors, sample sizes, sTg measurement methods, TNM staging, recombinant human TSH use, and threshold definitions^2,3,16–19^.

Notably, previous studies are often limited by small sample sizes, selection bias, and variable statistical methodologies^1–3,20^. In our study, we integrated multiple clinical parameters, including the first ^131^I administered activity, and constructed a nomogram based on data from a large retrospective cohort. We excluded patients who died from non-thyroidal cancer, developed pulmonary metastases after ^131^I treatment, or were under 18 years of age to minimize statistical bias.

Our univariate and multivariate analyses showed pathological type, first ^131^I administrated activity, and ^131^I uptake as independent predictors of PFS. Effective ^131^I uptake was shown to be critical for achieving optimal outcomes. Furthermore, factors such as male sex, age ≥ 55 years, concomitant lung and other organ metastases, initial sTg levels of ≥ 500 ng/mL, and larger metastatic lung lesions were associated with poorer survival. Conversely, higher first ^131^I administrated activity was inversely associated with the risk of death. In our study, ^131^I uptake was an independent predictor of OS and PFS, aligning with previous research^2,7,8,16,21–23^. Tumors exhibiting secondary or consistent ^131^I uptake had significantly better survival compared to those with primary non-uptake. Primary resistance to ^131^I was observed in cases with no uptake ^131^I during the first treatment and in those with persistent progression despite uptake. Secondary resistance, characterized by the loss of uptake after subsequent treatment, may occur due to insufficient radiation doses failing to eliminate tumor cells^24^. However, these cases could not be precisely identified from our data. Advanced age and male gender have been reported as negative prognostic factors^7,8^. Additionally, Tg levels serve as a biomarker for tumor burden in DTC, showing a linear relationship with primary tumor size, lymph node involvement, and distant metastases^25^. Previous studies have consistently demonstrated a correlation between high Tg levels at the time of ^131^I ablation and poor prognosis^17^.

In cohorts where 68.8% of the patients had ^131^I-avid lesions, treatment achieved a cured rate between10–49% with an overall efficacy of 60.3%; efficacy increased to 84% in patients with high-functioning metastases^2,7,24,26^. These findings are consistent with our data, although cure rates vary widely across studies. In the present study, 6.6% of patients achieved a CR, with discrepancies potentially due to differences in staging, metastatic sites, and criteria for defining CR across different time periods. Studies on DTC with distant metastases have reported 5-year survival rates ranging from 68 to 86.8%, and 10-year survival rates extending up to 65.79% following ^131^I therapy^7,26,27^.

The administered ^131^I activity is influenced by various factors and is potentially key to prognosis. The range of first activity reported in studies varies widely, from ≤ 2.00 GBq to dosimetry-recommended levels of 2.66 to 18.5 GBq^2,3,27,28^. However, there is a lack of high-quality evidence linking first ^131^I activity with outcomes in patients with distant metastases. One large study indicated that low remnant ablation activity was associated with increased DTC-specific mortality and recurrence rates, particularly in high-risk aged ≥ 45 years with M0 disease, but not in those with M1 disease (136 cases)^28^. Another study comparing dosimetric and empirical treatments suggested a trend toward better PFS with higher activity at first activity, though no significant survival differences were observed in patients with distant metastases, possibly due to the small sample size^29^. Our study demonstrates a significant association between higher initial ^131^I activity and improved OS and PFS. However, evidence supporting the mechanisms behind this association remains limited^28,29^. Potential mechanisms for the adverse impact of lower activity include non-tumoricidal doses leading to tumor dedifferentiation, epigenetic changes, stunning effects, and other processes^20,30–32^. Further research is necessary to elucidate these mechanisms. Studies have shown that the absorbed dose rarely exceeds 20 Gy after ≥ 4 treatments with activities ranging from 3.70 to 7.40 GBq (100–200 mCi)^24^. Thus, each treatment opportunity should aim for maximum efficacy, although clinical evidence supporting this approach remains scarce^10,12,14,33^.

The nomogram developed in this study serves as an effective statistical model for prognostic visualization, highlighting factors that impact survival and predicting individual survival probabilities. Univariate and multivariate Cox regression analyses identified key determinants of OS and PFS in patients with distant metastases treated with ^131^I. The nomogram-predicted PFS and OS closely aligned with observed values on calibration plots, and C-index values were similar between training and validation cohorts, indicating strong discriminative ability and calibration accuracy. Significant differences were observed between high- and low-risk groups (3-, 5-, and 10-year OS and PFS), demonstrating the robust discriminatory power of our model. By integrating modifiable factors, such as initial ^131^I activity, our nomogram provides personalized and continuous risk assessments, offering actionable insights that could potentially enhance patient outcomes—insights that are not fully captured by traditional models^1–3^.

This study has some limitations. The single-center retrospective design and attrition during follow-up may have introduced selection bias. In addition, the lung metastases were not pathologically confirmed; the diagnosis was based on CT and sTg levels. Finally, comprehensive driver gene testing was not available for this cohort, which may have limited the granularity of our findings.

## Conclusion

Most patients in this cohort exhibited ^131^I uptake in there lesions with many benefiting from ^131^I therapy and a subset potentially achieving a cure. The nomogram models developed in this study incorporated commonly used clinical and pathological parameters, with a unique emphasis on the activity of the first ^131^I treatment. Although this study has limitations, it provides valuable insights that could enhance clinical decision-making. Future prospective studies exploring the relationship between first ^131^I administrated activity and prognosis in patients with distant metastases could yield significant clinical insights.

## Materials and methods

### Patients

This single center retrospective study included patients with DTC who underwent ^131^I therapy at the Zhejiang Cancer Hospital between 2007 and 2020. Patients who underwent bilateral total or subtotal thyroidectomy for pathologically confirmed DTC, were aged over 18 years, completed standard ^131^I treatment, and had metastases to the lung (and other organs in some cases) were included. Those developing lung metastases after ^131^I treatment, dying from causes unrelated to thyroid cancer (including accidental death), demonstrating pathological evidence of undifferentiated thyroid cancer (or components of undifferentiated cancer), and aged under 18 years were excluded. The presence of lung and other distant metastases was diagnosed and confirmed using at least one of the following approaches: clinical manifestations, comprehensive imaging (including computed tomography [CT], magnetic resonance imaging [MRI], and/or 18 F-fluorodeoxyglucose positron emission tomography/CT [18 F-FDG-PET/CT]), and thyroglobulin (Tg) levels; surgical biopsy (for distant metastatic lesions) with pathological confirmation of thyroid origin; and ^131^I whole-body scans showing evidence of metastatic lesions. Data pertaining to clinical characteristics at presentation and during follow-up were obtained from the medical records. The Ethics Committee of the Zhejiang Cancer Hospital provided ethical approval for this study (IRB − 2024-53), which was performed in accordance with the principles of the Declaration of Helsinki.

### Treatment

Prior to treatment with ^131^I, thyroid hormone was withdrawn for at least three weeks and a low-iodine diet was advised for more than two weeks. Imaging examinations, including chest CT, neck ultrasonography, and whole-body bone imaging, were performed. Additional assessment, including PET-CT and MRI, were performed as needed to evaluate the lesion burden and stage. The levels of thyroid-stimulating hormone (TSH) and Tg were also evaluated. The activity of administered ^131^I was determined according to the empiric fixed activity approach recommended by the guidelines, that considers factors such as patient age, body weight, and tumor burden^10,13,14,33^. Thyroid hormone suppression therapy was initiated two days after the administration of ^131^I therapy. A post-therapeutic whole-body single-photon emission computed tomography (SPECT, alone or with CT) scan was performed 3–4 days after treatment to assess ^131^I uptake and lesion distribution. All tumors were staged based on the 8th edition of the American Joint Committee on Cancer-Tumor, Node, Metastasis classification^34^.

### Evaluation of therapeutic efficacy

The efficacy of ^131^I treatment was evaluated based on imaging (CT and other modalities) and TSH-stimulated Tg (sTg) levels (after thyroid hormone withdrawal for > 3 weeks). CT-based evaluation was performed using Response Evaluation Criteria in Solid Tumors (version 1.1); 2 radiologists (each with more than 10 years of experience) assessed the scans. A decrease or increase in sTg levels by > 25% was considered to represent partial response (PR)^35^ or progressive disease (PD), respectively, whereas any decrease or increase within 25% was considered to indicate stable disease (SD). In cases demonstrating inconsistency between sTg levels and CT findings, the latter were used to determine efficacy. PR was confirmed by the absence of structural or functional disease on post-treatment imaging and suppressed Tg and sTg levels of ≥ 1 µg/L and ≥ 10 µg/L, respectively. Conversely, CR was confirmed in cases showing no evidence of structural or functional disease on post-treatment imaging and suppressed Tg and sTg levels of ≤ 1 µg/L and ≤ 10 µg/L, respectively.

### Statistical analysis

All statistical analyses were performed using R software (version 4.3.2). The subjects were randomly divided into training (70%) and validation (30%) sets using the sample function in R. The training set was used to establish a predictive model for the efficacy of ^131^I treatment, while the validation set was used for model validation. Categorical variables were described by frequency (%), and comparisons between groups were made using the Chi-square or Fisher’s exact tests.

For predictive model construction, univariate Cox regression was first performed to identify potential predictive factors; overall survival (OS) and progression-free survival (PFS) were considered as dependent variables (*p* < 0.05). OS was defined as the time from treatment initiation to death from any cause, and PFS was defined as the time from treatment initiation to progression or death. Multivariate Cox regression analysis (stepwise and bidirectional) was then performed to identify independent predictive factors (*p* < 0.05). A nomogram was constructed based on these factors and validated using bootstrap sampling and calibration curves. Time-dependent receiver operating characteristics curve analyses were performed to calculate the C-index and other indicators.

The constructed nomogram model was validated using data from the validation set, and nomogram scores were calculated for all study subjects. The optimal cutoff value for the scores was determined using the surv_cutpoint function from the survminer R package; high and low-risk groups were defined based on this cutoff. Kaplan-Meier curves were plotted for the training and validation sets and the entire population, and the model was validated using the log-rank test.

The rms package was used for constructing and plotting the nomograms, and the riskRegression, ggprism, and ggplot2 packages were used for drawing time-dependent receiver operating characteristics curves. The survminer package was used for survival analysis. All tests were two-sided, and a *p*-value of < 0.05 was considered statistically significant.

## Electronic supplementary material

Below is the link to the electronic supplementary material.

Supplementary Material 1

## Data Availability

The data generated in the present study may be requested from the corresponding author.
