# Supplementary material for: Nomogram models for predicting outcomes in thyroid cancer patients with distant metastasis receiving 131iodine therapy
Source: Sci Rep. 2025 Jan 20;15:2486. doi: 10.1038/s41598-025-86169-7 (PMC11747094; doi:10.1038/s41598-025-86169-7)
Supplement: Supplementary file 1 — Supplementary Material 1 [file 41598_2025_86169_MOESM1_ESM.pdf]

Group	Time Point	OS	95% CI Lower Bound	95% CI Upper Bound	PFS	95% CI Lower Bound	95% CI Upper Bound
training set-low-risk	3year	93.90%	89.30%	98.80%	82.30%	76.90%	88.00%
training set-low-risk	5year	91.30%	85.60%	97.30%	81.40%	75.80%	87.40%
training set-low-risk	10year	76.90%	66.40%	89.10%	81.40%	75.80%	87.40%
training set-high-risk	3year	67.70%	46.00%	99.70%	59.30%	50.60%	69.50%
training set-high-risk	5year	67.70%	46.00%	99.70%	55.50%	46.60%	66.20%
training set-high-risk	10year	56.40%	33.30%	95.60%	53.80%	44.70%	64.80%
validation set-low-risk	3year	99.50%	98.50%	100.00%	80.50%	72.10%	89.90%
validation set-low-risk	5year	98.20%	96.10%	100.00%	80.50%	72.10%	89.90%
validation set-low-risk	10year	90.70%	84.90%	96.80%	80.50%	72.10%	89.90%
validation set-high-risk	3year	83.50%	72.20%	96.50%	50.90%	38.40%	67.50%
validation set-high-risk	5year	65.90%	51.10%	85.00%	47.90%	35.30%	65.00%
validation set-high-risk	10year	31.70%	17.00%	59.00%	47.90%	35.30%	65.00%
complete set-low-risk	3year	97.70%	96.10%	99.40%	81.70%	77.20%	86.60%
complete set-low-risk	5year	96.00%	93.70%	98.40%	81.10%	76.50%	86.10%
complete set-low-risk	10year	86.20%	80.80%	91.90%	81.10%	76.50%	86.10%
complete set-high-risk	3year	79.50%	68.90%	91.80%	56.70%	49.40%	65.20%
complete set-high-risk	5year	66.30%	53.40%	82.20%	53.20%	45.60%	62.00%
complete set-high-risk	10year	37.40%	23.40%	59.90%	51.90%	44.30%	60.90%
